# Evaluation of the proliferation marker Ki-67 in a large prostatectomy cohort

**DOI:** 10.1371/journal.pone.0186852

**Published:** 2017-11-15

**Authors:** Elin Richardsen, Sigve Andersen, Samer Al-Saad, Mehrdad Rakaee, Yngve Nordby, Mona Irene Pedersen, Nora Ness, Thea Grindstad, Ingeborg Movik, Tom Dønnem, Roy Bremnes, Lill-Tove Busund

**Affiliations:** 1 Translational Cancer Research Group, Department of Medical Biology, UiT The Arctic University of Norway, Tromso, North Norway; 2 Department of Clinical Pathology, University Hospital of North Norway, Tromso, North Norway; 3 Translational Cancer Research Group, Department of Medical Biology, UiT The Arctic University of Norway, Tromsø, Tromso, North Norway; 4 Department of Oncology, University Hospital of North Norway, Tromso, North Norway; 5 Department of Urology, University Hospital of North Norway, Tromso, North Norway; King's College London, UNITED KINGDOM

## Abstract

The tumor proliferation index marker Ki-67 is strongly associated with tumor cell proliferation, growth and progression, and is widely used in routine clinicopathological investigation. Prostate cancer is a complex multifaceted and biologically heterogeneous disease, and overtreatment of localized, low volume indolent tumors, is evident. Here, we aimed to assess Ki-67 expression and related outcomes of 535 patients treated with radical prostatectomy. The percentage of tumor epithelial cells expressing Ki-67 was determined by immunohistochemical assay, both digital image analysis and visual scoring by light microscope were used for quantification. The association of Ki-67 and prostate cancer was evaluated, as well as its prognostic value. There was a positive correlation between high expression of Ki-67 and Gleason score > 7 (p < 0.001) as well as tumor size (≥ 20 mm, p = 0.03). In univariate analyses, a high expression of Ki-67 in tumor epithelium was significantly associated with biochemical failure (BF) (digital scoring, p = 0.014) and (visual scoring, p = 0.004). In the multivariate analyses, a high level of Ki-67 was an independent poor prognostic factor for biochemical failure-free survival (BFFS) (Visual scoring, Ki67, p = 0.012, HR:1.50, CI95% 1.10–2.06). In conclusion, high Ki-67 expression is an independent negative prognostic marker for biochemical failure. Our findings support the role of Ki-67 as a significant, poor prognostic factor for in prostate cancer outcome.

## Introduction

Radical prostatectomy as a primary treatment for clinically localized prostate cancer (PC) has increased dramatically over the past decade due to prostate specific antigen (PSA) screening [1]. Despite increased ability to detect cancer, the clinical behavior of PC remains hard to predict as it ranges from indolent to highly aggressive tumors [2]. Therefore, new prognostic biomarkers are urgently needed. Management of PC today relies largely on standard clinical factors including Gleason score, prostate specific antigen (PSA) level, clinical stage and measures of tumor extent on biopsy and imaging. These methods, however, have a rather limited potential to stratify indolent from aggressive disease [2–3]. After localized radical prostatectomy and radiation therapy, 20–40% of the patients will relapse, progress, and will be in need of androgen deprivation therapies [4].

The proliferation marker Ki-67 reflects the tumor cell proliferation rate as it correlates with progression, metastasis and prognosis in a number of different malignancies [5–9]. Ki-67 is a nuclear cell cycle-associated regulatory protein and the expression of it can be detected during the interphase in the nucleus of tumor epithelial cells [10]. The fact that Ki-67 is involved during all active phases of the cell cycle (G1, S, G2 and mitosis), and absent in resting cells (G0 phase), has made it an excellent marker for determining tumor growth fraction [11]. The Ki-67 (MIB-1 antibody) labeling index is the best studied PC marker in needle-biopsies up to date [12–17]. Several have found that Ki-67 labeling index shows strong correlation with Gleason score in diagnostic biopsies [12, 14], in subsequent radical prostatectomy [15–17], or both [13]. Others have found Ki-67 to be a biomarker for disease-free survival [13], seminal vesicle invasion and postoperative biochemical recurrence [17], and cancer specific death after radical prostatectomy [18]. Others have not been able to confirm these results [16].

The prognostic value of Ki-67 in PC remains somewhat contradictory and inconclusive mainly due to the biologic tumor heterogeneity, lack of standardization in the immunohistochemical (IHC) assays, quantification methods, cutoff-points used for risk classification, and intra- and inter-observer variability.

The objective of this large multicenter study with long follow-up was to investigate if Ki-67 may provide additional information to prognostic indicators in PC. A cohort of 535 PC patients, treated with radical prostatectomies but without pre-operative hormonal therapy was investigated.

## Materials and methods

### Patients and tissue microarray

671 patients with radical prostatectomies (RPs) diagnosed with adenocarcinoma of the prostate were retrospectively identified. The samples were collected between 01.01.1995 to 31.12.2005 from the archives of the Departments of Clinical Pathology at the University Hospital of North Norway, St. Olav Hospital and Nordland Hospital. Of these, 136 patients were excluded due to other cancer within five years of PC diagnosis, radiotherapy to the pelvis prior to surgery, inadequate paraffin-embedded tissue blocks and missing follow-up data. None of the patients received a preoperative hormonal therapy. Included in the study was a total of 535 patients with complete follow-up data and available PC tissue. Median follow-up was 12.4 years (range 1.5–20 years). The most recent follow-up was December 2015. Biochemical failure (BF) was defined as postoperative PSA ≥ 0.4ng/ml and rising in a minimum of two different blood samples. Biochemical failure-free survival (BFFS) was calculated as time from surgery to last follow up (FU) date, or date with PSA above threshold (≥ 0.4 ng/ml in a minimum of two different blood samples postoperatively). Clinical failure-free survival (CFFS) was defined as verified, symptomatic, locally advanced progression after radical treatments or metastasis to bone, visceral organs or lymph nodes verified by radiology. Prostate cancer death free survival (PCDFS), was defined as death caused by PC stated in the patient’s journal. Further information regarding patients' data, exclusion criteria, definitions of variables and endpoints, has been previously published [19]. All primary cancers were histologically reviewed by two pathologists (ER and LTB) and the tumors were graded according to the recent Gleason grading system; *The 2014 International Society of Urological Pathology (ISUP) Consensus Conference on Gleason Grading of Prostatic Carcinoma* [20] and staged according to the new guidelines [21].

The current study was approved by the ethics committee, REK Nord (2009/1393), including a mandatory re-application January 22. 2016, and the Data Protection Official for Research. The National Data Inspection Board have approved the study. The ethics committee waived the need for patients consent in this retrospective study. The patient records were anonymized prior the research. The reporting of clinicopathological variables, survival data and biomarker expressions was conducted in accordance with the REMARK guidelines [22].

### Microarray construction

We used tissue microarrays (TMA) and twelve TMA blocks were constructed. A tissue-arraying instrument (Beecher Instruments, Silver Springs, MD, USA) was used for this purpose. We collected formalin-fixed paraffin-embedded (FFPE) tissue blocks from included patients. The author (ER) identified two different areas of tumor compartment (tumor epithelial cells) and two areas of tumor surrounding microenvironment. Two areas of normal epithelial cells and normal stromal tissue was also sampled as controls. Cores with a diameter of 0.6 mm from donor block was collected and inserted into recipient TMA blocks. Multiple 4 μm sections were cut with a Micron microtome (HM355S), affixed to glass slides, and sealed with paraffin. The detailed methodology has been reported previously [23].

### Immunohistochemistry (IHC) and quantification of Ki67 immunostaining

The following antibody from Ventana Medical Systems (Tucson, Arizona, USA) was applied to assess the proliferative activity of normal and neoplastic tissues: CONFIRM Ki-67 (clone, 30–9), a rabbit monoclonal primary antibody directed against the C-terminal portion of the Ki-67 antigen. The applied antibody is used in routine diagnostic IHC and has FDA approval (510k) for IVD (*in vitro* diagnostic) use. Ki-67 positive staining was identified by the presence of brown nuclear (DAB) staining in tumor cells. Ki-67 index was quantified using The ARIOL imaging system (Applied Imaging Corp., San Jose, CA, USA) and light microscope. The ARIOL imaging system consisted of a microscope (Olympus BX 61), an automatic stage, slide loader and a camera. All cores were photographed at 20x magnification and the images was semi-quantitatively scored. For both methods, the ARIOL imaging system and the light microscope, the percentage of positive nuclear stained tumor cells among total number of at least 200 tumor cells were counted for each core and scored according to the following system: 0 = 0%, 1 = 1–2.5%, 2 = 2.6–4, 3 ≥ 5%. For both methods, the scoring values were then dichotomized as high or low expression separated by mean value. A high expression was defined as scoring values ≥ 1.43 (visual scoring using light microscope) and ≥ 1.34 (digital scoring).

### Statistical methods

All statistical analyses were performed using the statistical package IBM SPSS, version 24 (SPSS Inc., Chicago, IL, USA). Spearman correlation coefficient was used to examine the association between Ki-67 score and clinicopathological variables. The Kaplan-Meier method was used for the univariate survival analysis, and log-rank test was used to assess statistical significance. Univariate analyses were performed for the following end-points: biochemical failure (BF), clinical failure (CF) and death of prostate cancer (PCD). All significant variables from the univariate analyses were entered into the multivariate model using backward stepwise Cox regression model with a probability for stepwise entry and removal at 0.05 and 0.1, respectively. The IHC scoring values from each pathologist were compared for inter-observer reliability by use of a two-way random effect model with absolute agreement definition. The significance level used was p < 0.05 for all analyses.

## Results

### Patient characteristics

Overview of the patient’s characteristics’ is presented in Table 1 and S1 Table. Median age at surgery was 62 years (47 to 76). The surgical procedures were retropubic in 435 cases (81%) and perineal in 100 cases (19%). Gleason grade group ranged from 1 to 5 (updated Gleason grade system); 1 (≤ 6), 2 (3+4), 3 (4+3), 4 (4+4) and 5 (≥ 8). Tumor stage ranged from T2a to T3b. Median PSA was 8.8 (range 0.7–104). At the last follow-up, 200 (37%) had BF, 56 (11%) had experienced CF and 18 (3.4%) had died of PC.

**Table 1:** Patient characteristics clinicopathological variables and their prognostic variables for BF, CF and PCD (univariate analysis; log-rank test) (N = 535).

Characteristic	Patients (n)	Patients (%)	BF (200 events)		CF (56 events)		PCD (18 events)	
			5-year EFS (%)	p	10-year EFS (%)	p	10-year EFS (%)	p
**Age**				0.237		**0.038**		0.600
≤ 65 years	357	67	76		92		97	
> 65 years	178	33	70		88		96	
**pT-stage**				**<0.001**		**<0.001**		**0.027**
pT2	374	70	83		97		98	
pT3a	114	21	61		87		98	
pT3b	47	9	43		73		89	
**Preoperative PSA**				**<0.001**		0.029		0.061
PSA<10	308	57	81		95		99	
PSA>10	221	42	68		89		95	
Missing	6	1	-		-		-	
**ISUP grade group**				**<0.001**		**<0.001**		**<0.001**
1 (3+3)	183	34	83		98		99	
2 (3+4)	219	41	77		94		98	
3 (4+3)	81	15	70		90		95	
4 (4+4)	17	4	58		86		94	
5 (>8)	35	6	37		65		87	
**Tumor Size**				**<0.001**		**0.019**		0.098
0–20 mm	250	47	83		94		99	
>20 mm	285	53	68		88		96	
**PNI**				**<0.001**		**<0.001**		**0.002**
No	401	75	80		96		98	
Yes	134	25	60		83		93	
**PSM**				**0.049**		**0.198**		0.697
No	249	47	81		96		97	
Yes	286	53	69		90		97	
**Non-apical PSM**				**<0.001**		**<0.001**		**0.029**
No	381	71	82		96		98	
Yes	154	29	57		85		94	
**Apical PSM**				0.063		0.427		0.313
No	325	61	74		90		96	
Yes	210	39	77		92		98	
**Vascular infiltration**				**<0.001**		**<0.001**		**0.009**
No	492	92	77		95		98	
Yes	43	8	47		69		88	
**Nstage**				**<0.001**		**<0.001**		**<0.001**
Nx	264	49	79		96		99	
N0	268	50	72		90		97	
N1	3	1	0		33		67	

Abbreviations: BF = biochemical failure; CF = clinical failure; EFS = event free survival in months; PCD = prostate cancer death; p = p value for log rank statistics for difference in event free survival; PC = prostate cancer; PNI = perineural infiltration; PSA = prostate specific antigen; PSM = positive surgical margin; Nstage = nodal status

### Ki67 expression and correlations with clinicopathological variables

**Fig 1:**
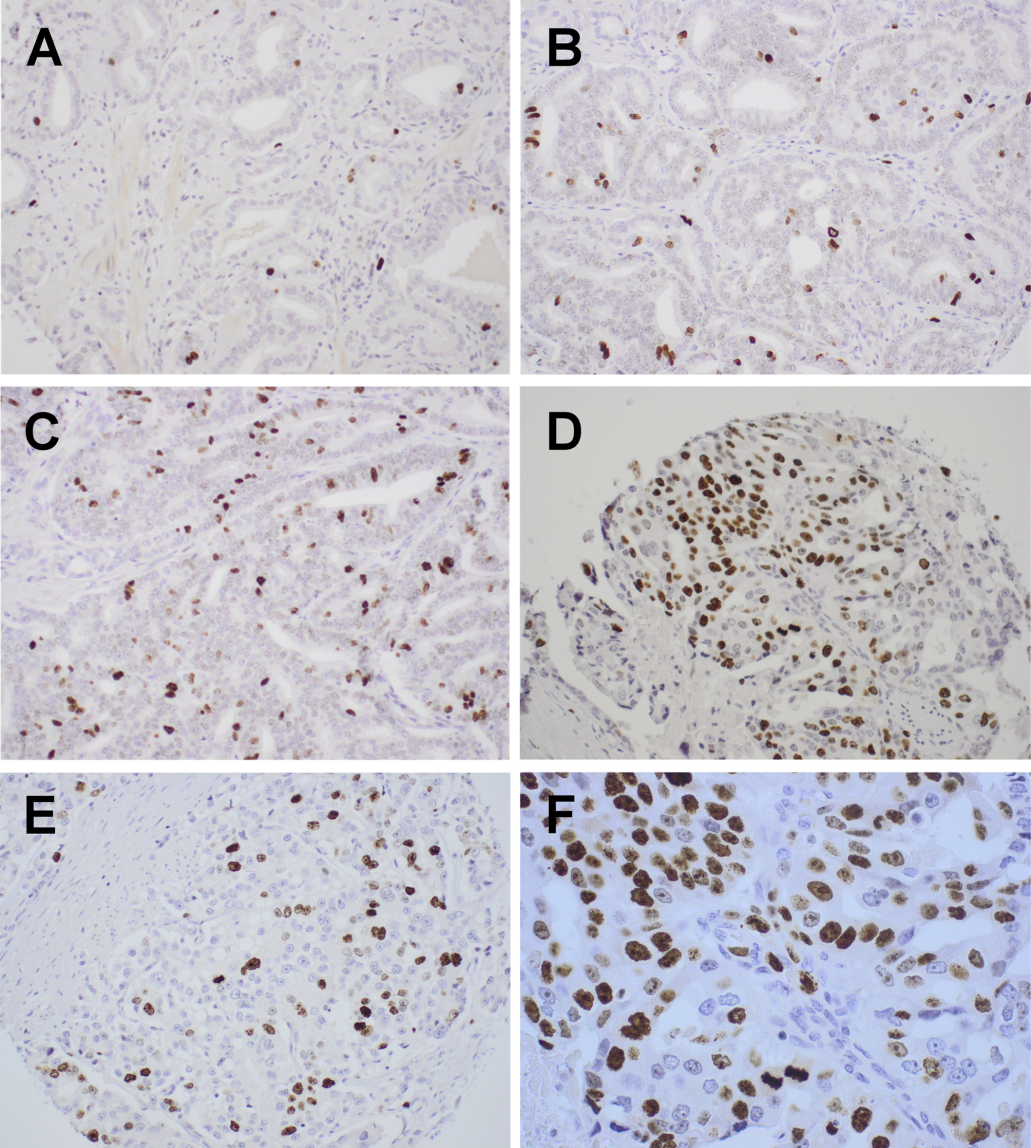
Representative immunohistochemical staining for Ki-67. **(A)** Gleason grade group 1 (3+3) with Ki-67 <1–2.5%; **(B)** Gleason grade group 3 (4+3) with Ki-67 <1–2.5%; **(C)** Gleason grade group 3 (4+3) with Ki-67 2.6–4%; **(D)** Gleason grade group 4 (4+4) with Ki-67 ≥ 5; **(E)** Gleason grade group 5 (4–5) with Ki-67 2.6–4; **(F)** Picture showing the different Ki-67 expression of nuclear staining, varying from negative to strong.

Nuclear staining of Ki-67 in tumor epithelial cells was observed. The intensity of the nuclear staining was varying from negative, weak, to moderate and strong, and all grades, except negative results, were regarded as positive cells (Fig 1). Ki-67 nuclear staining was evaluated by using light microscope (visual scoring) and digital image analysis. Two experienced pathologists independently scored the TMA-slides without any prior knowledge of the patients’ clinicopathological data or any clinical end-points. Positive Ki-67 staining was clearly detected in the nucleus of tumor epithelial cells, in 452 (84%) of the total of 535 patients (visual scoring) and in 483 (90%), digital scoring method. Of those with detected staining, 61% had a low Ki-67 expression (< 1.43) and 39% had a high expression (≥ 1.43). Using cutoff-value < 1.34 and ≥ 1.34, 60% had low expression and 40% a high expression, respectively (Fig 1A–1F). Interobserver scoring agreement (ICC), for Ki-67 expression in tumor epithelial cells was: ICC = 0.78 (CI: 0.74–0.82, p < 0.001). When stratifying the analyses by the different surgical centres, the results remained unchanged. (S1 Fig)

The correlation between Ki-67 level and clinicopathological variables was generally weak or non-significant (r < 0.2). However, positive correlations were found between high Ki-67 expression and Gleason grade ≥8 (p = 0.001), tumor size ≥ 20 millimeter (p = 0.03) and pT-Stage T3b (p = 0.053).

We also correlated Ki-67 expression with previous investigated markers [24–27], but no significant correlation was found.

#### Univariate analyses

**Fig 2:**
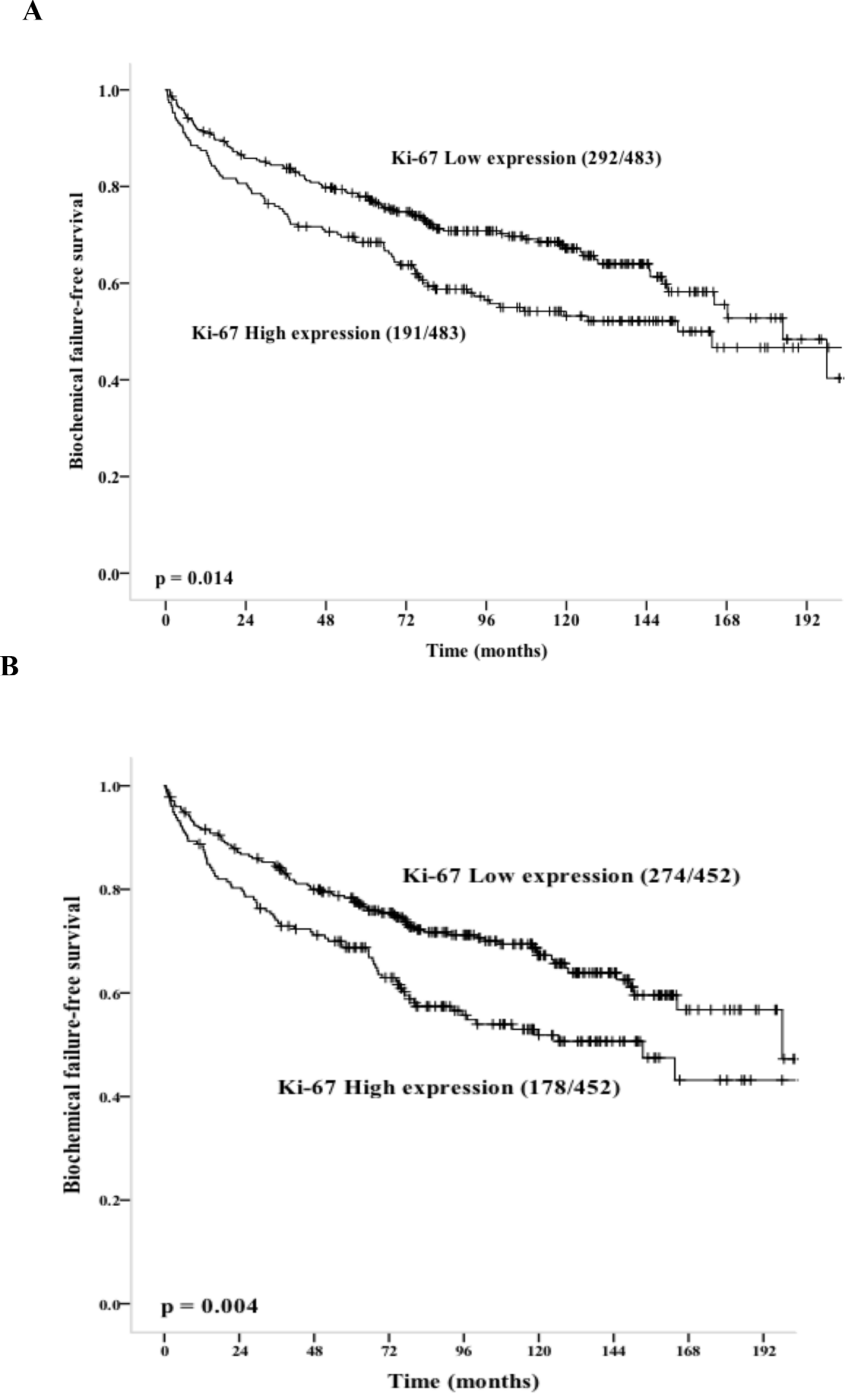
Survival analysis for Ki-67 and mitotic count in primary tumors. (Kaplan-Meier method). Number of events/number of cases are given in parenthesis. **(A)** Digital scoring; **(B)** Visual scoring.

Significant variables, for the endpoints BF, CF and PCD are all presented in Table 1. For BF, significant prognostic factors were: pT-stage (p < 0.001), preoperative PSA (p < 0.001), Gleason score (p < 0.001), tumor size (p < 0.001), perineural infiltration (PNI, p < 0.001), non-apical PSM (p  =  0.049), apical PSM (p < 0.001), vascular infiltration (p < 0.001), and pN-stage (p < 0.001). For CF, significant prognostic factors were: pT-stage (p < 0.001), Gleason score (p < 0.001), tumor size (p  =  0.019), PNI (p  =  0.001), non-apical PSM (p < 0.001), vascular infiltration (p < 0.001) and pN-stage (p < 0.001). For PCD the significant prognostic factors were: pT-stage (p  =  0.027), Gleason score (p < 0.001), PNI (p  =  0.002), non-apical PSM (p  =  0.029), vascular infiltration (p  =  0.009) and pN-stage (p < 0.001). A high Ki-67 expression (≥ 1.34) in tumor epithelium (digital scoring method) was significantly associated with BF, (p = 0.014, Fig 2A), but not with CF (p = 0.405) or PCD (p = 0.752). For visual scoring with high cut-off value ≥ 1.43, high Ki-67 expression was significant with BF (p = 0.04), but not with CF (p = 0.129) or PCD (p = 0.502). Stratification of the cohort into prognostic groups according to the American Joint Committee on Cancer (AJCC) TNM system was implemented [28]. Neither high- or low levels of Ki-67 was significant for any the staging groups; I (n = 43), IIA (n = 111), IIB (219), III (n = 219) or IV (n = 3).

#### Multivariate analyses

Results from the multivariate analysis are represented in Table 2 (digital scoring): pT-stage (p = 0.003), pT3a (p = 0.001, HR: 0.44, CI95% 0.27–0.82), ISUP grade group 2 (p = 0.028, HR: 0.52, CI95% 0.30–0.93), PNI (p = 0.040, HR: 1.70, CI95% 0.50–0.98) and non-apical PSM (p = 0.002, HR: 0.60, CI95% 0.43–0.83) were independent prognostic factors for BF. For CF, ISUP grade group 3 (p < 0.000), ISUP grade group 2 (< 0.000, HR: 0.08, CI95% 0.03–0.23); ISUP grade group 3 (p = 0.001, HR: 0.28 CI95% 0.13–0.58), ISUP grade group 4 (p = 0.022, HR: 0.38 CI95% 0.17–0.87) and non-apical PSM (p = 0.041, HR: 0.60, CI95% 0.54–0.97). For PCD, ISUP grade group 2 (p = 0.033, HR: 0.11, CI95% 0.03–0.86), ISUP grade group 3 (p = 0.035, HR:0.19, CI95% 0.04–0.89) and PNI (p = 0.027, HR: 0.30, CI95% 0.11–0.89–9). Ki-67 was not significant with none of the endpoints. Visual scoring is presented in Table 3. For BF, pT-stage (p < 0.002), pT3a (p < 0.000, HR: 0.42, CI95% 0.26–0.69), pT3b (p = 0.047, HR: 0.62, CI95% 0.39–0.99), preoperative PSA > 10ng/ml (p = 0.046, HR: 0.68, CI95% 0.49–0.94), PNI (p < 0.008, HR: 0.62, CI95% 0.44–0.88), non-apical PSM (p < 0.000, HR: 0.54, CI95% 0.39–0.77), apical PSM (p = 0.043, HR: 1.42, CI95% 1.01–2.00) and Ki67 (p = 0.012, HR: 1.50, CI95% 1.10–2.06). For CF, ISUP grade group (p < 0.000), grade group 2 (p < 0.000, HR: 0.07, CI95% 0.02–0.21), ISUP grade group 3 (p < 0.000, HR: 0.25, CI95% 0.12–0.51), ISUP grade group 4 (p = 0.015, HR: 0.34, CI95% 0.15–1.30), non-apical PSM (p = 0.007, HR: 0.45, CI95% 0.30–1.10). For PCD, PSA > 10ng/ml (p = 0.034, HR: 0.25, CI95% 0.07–0.90), ISUP grade group 2 (p = 0.047, HR: 0.17, CI95% 0.07–0.90) and ISUP grade group 3 (p = 0.035, HR: 0.20, CI95% 0.04–0.91). Ki-67 was not significant with endpoints CF or PCD.

**Table 2:** Multivariate analyses (Cox regression, backward conditional) of Ki67 levels and significant clinicopathological variables. (n = 535). Digital scoring method.

Characteristic	No	BF (200 events) HR CI95% p	CF (56 events) HR CI95% p	PCD (18 events) HR CI95% p
	
**Age**		NS	NS	NS
≤ 65 years	321			
> 65 years	65			
**pT-stage**		**0.003**	NS	NS
pT2	333	1		
pT3a	102	0.44 0.27–0.82 **0.003**		
pT3b	40	0.70 0.44–1.12 0.140		
**Preoperative PSA**		NS	NS	NS
PSA <10	278			
PSA >10	200			
**ISUP grade group**		0.063	**0.000**	0.095
1 (3+3)	157	1	1	1
2 (3+4)	198	0.52 0.30–0.93 **0.028**	0.08 0.03–0.23 **0.000**	0.11 0.03–0.86 **0.033**
3 (4+3)	74	0.62 0.35–1.08 0.091	0.28 0.13–0.58 **0.001**	0.19 0.04–0.89 **0.035**
4 (4+4)	17	0.87 0.48–1.56 0.633	0.38 0.17–0.87 **0.022**	0.79 0.25–2.65 0.700
5 (>8)	32	1.02 0.46–2.26 0.956	0.44 0.12–1.54 0.199	0.61 0.07–5.31 0.651
**Tumor size**		NS	NS	NS
0–20 mm	215			
>20 mm	263			
**PNI**		**0.040**	NS	**0.027**
No	401	1		1
Yes	134	0.70 0.50–0.98		0.30 0.11–0.87
**Non-apical PSM**		**0.002**	**0.041**	NS
No	381	1	1	
Yes	154	0.60 0.43–0.83	0.54 0.30–0.97	
**Apical PSM**		NS	NS	NS
No	325			
Yes	210			
**Vascular infiltration**		NS	NS	NS
No	437			
Yes	41			
**Ki67 level**		NS	NS	NS
Low	289			
High	189			

Abbreviations: BF = biochemical failure; CF = clinical failure; PSA = prostate specific antigen; PNI = perineural infiltration, PSM = positive surgical margin; NS = not significant.

**Table 3:** Multivariate analyses (Cox regression, backward conditional) of Ki67 levels and significant clinicopathological variables. (n = 535). Visual scoring method.

Characteristic	No	BF (200 events) HR CI95% p	CF (56 events) HR CI95% p	PCD (18 events) HR CI95% p
	
**Age**		NS	NS	NS
≤ 65 years	299			
> 65 years	148			
**pT-stage**		**0.002**	NS	0.090
pT2	307	1		1
pT3a	102	0.42 0.26–0.69 **0.001**		0.07 0.01–0.79 **0.031**
pT3b	38	0.62 0.39–0.99 **0.047**		0.11 0.01–0.96 **0.046**
**Preoperative PSA**		**0.046**	NS	**0.034**
PSA <10	255	1		1
PSA >10	192	0.62 0.49–0.94		0.25 0.07–0.90
**ISUP grade group**		0.077	**0.000**	0.161
1 (3+3)	139	1	1	1
2 (3+4)	190	0.00 0.00–0.47 **0.023**	0.07 0.02–0.21 **0.000**	0.17 0.03–0.98 **0.047**
3 (4+3)	71	0.02 0.00–0.54 **0.020**	0.25 0.12–0.51 **0.000**	0.13 0.04–0.91 **0.037**
4 (4+4)	16	0.03 0.00–0.78 **0.035**	0.34 0.15–1.30 **0.015**	0.66 0.18–2.33 0.513
5 (≥9)	31	0.18 0.23–1.11 0.065	0.37 0.11–1.30 **0.121**	0.59 0.07–5.17 0.631
**Tumor size**		NS	NS	NS
0–20 mm	192			
>20 mm	255			
**PNI**		**0.008**	NS	**0.005**
No	332	1		1
Yes	115	0.62 0.44–0.88		0.19 0.05–0.66
**Non-apical PSM**		**0.000**	**0.007**	
No	313	1	1	
Yes	134	0.54 0.39–0.77	0.45 0.30–1.10	
**Apical PSM**		**0.034**	NS	NS
No	274	1		
Yes	173	1.42 1.01–2.00		
**Vascular infiltration**		NS	NS	NS
No	408			
Yes	39			
**Ki67 level**		**0.012**	NS 1	NS
Low	257	1		
High	175	1.50 1.10–1.26		

**Abbreviations:** BF = biochemical failure; CF = clinical failure; PSA = prostate specific antigen; PNI = perineural infiltration, PSM = positive surgical margin; NS = not significant.

## Discussion

Uncontrolled proliferation is a hallmark of malignancy and the measurement of Ki-67 antigen by using IHC is the most widely performed assessment of a tumor’s proliferation potential. In this large-scale multi-center study with long-term survival data, we found that a high expression of Ki-67 was an independent predictor for biochemical failure (both scoring methods). By using visual scorings method, a high Ki-67 was found as an independent predictor for BF in multivariate analyses. High expression of Ki-67 was strongly correlated to Gleason grade ≥ 8 and increased tumor size (> 20 mm). To the best of our knowledge, our study is one of the largest series to explore the prognostic impact of mitotic count by using two different methods.

Counting of mitoses is the classical method used to determine proliferative activity in normal and neoplastic tissues by using light microscopy. Despite that a lot of previous studies have confirmed Ki-67 as a prognostic factor for PC [12–18, 29–31], Ki-67 count has not been implemented in PC is not implemented in PC diagnosis. One of the main reason for this, includes, the morphologic heterogeneity of PC, as these methods only registers the M phase of the cell cycle [2–3] while, the number of identifiable mitoses may also depend upon the period of time between surgical removal and fixation of the specimen [32], Furthermore, there are several available antibodies or Ki-67 IHC staining, but there is no standard operating protocol, and the cut-off definition values for Ki-67 levels have not been established [33]. Moreover, the biological heterogeneity of Ki67 staining can occur across prostate cancer specimens, and definition of the location and extent of the area of the cancer that should be scored is controversial needs to be more clearly defined. This has the main important reason of the low interobserver reproducibility. Importantly, most studies are retrospectively designed with various number of patients included [12–18] which may explain the poor reproducibility of mitotic counts.

However, a significant association have been found between Ki-67 antigen expression and time to progression, high Gleason grade, large tumor size, metastasis, mortality and to predict distant metastases in men treated with radiotherapy and androgen deprivation [12, 13, 17, 18, 29–31, 34]. We did not find any association between Ki-67 and CF and PCD. This is most likely due with the low number of events for CF and PCD in our cohort. In two published PC studies measuring proliferating by using MIB-1 the investigators were not able to find significant association with PC and Ki-67 expression [15, 16].

Immunostaining for Ki-67 (IHC), is relatively straightforward. By means of immunostaining it is possible to assess the growth fraction of neoplastic cell populations. In this study, we used the anti-Ki-67 (clone:30–9) antibody which is a rabbit monoclonal primary antibody from Ventana. This antibody is intended for use to identify stained proliferating cells by light microscopy in sections of formalin fixed, paraffin embedded tissue. However, in practice, the monoclonal antibody MIB-1 is probably the most widely used proliferative marker. It reacts with an antigen that is only present in the nucleus of proliferating cells and has similar epitope sensitivity to Ki-67. There have been many reports of correlations between Ki-67 equivalent antibodies and other proliferation markers [35, 36]. The antibody used in this study is in daily use in our pathology department to assess the proliferative activity of normal and neoplastic cells. A study by Leonardo et al. [37] concluded that as a rabbit monoclonal antibody (RbMAb), it demonstrates increased sensitivity and strong specificity compared with mouse monoclonal antibodies (MMAbs). With intense nuclear staining and no adipose (K2) or cell membrane staining (MIB-1), CONFIRM Ki-67 (30–9) rabbit monoclonal antibody can be used in assessment of tumor aggressiveness [37].

Determination of proliferative activity by the use of Ki-67 depends on several factors, the most obvious being the interobserver variation. In our study, we used the same cut-off values and TMA-slides for both visual and digital analyses, and observed, in fact, a good ICC between the two investigators. At the other hand, the interpretation is not straightforward.

In the present study, we used two different approaches to measure Ki-67 expression, by visual and digital scoring. There was high inter-correlation agreement between the pathologist visual scoring and the pathologist digital scoring. This is in agreement with one similar study (no = 225) [37]. Limitations of our study include its retrospective nature as well as the use of TMA cores and not whole slide sections to determine the proliferative activity. Although the use of TMA may result in a bias, due to the heterogeneity of PC. A more representative method, at least with respect to Ki-67, could be better achieved with multiple cores taken from each single lesion. Another limitation might be the antibody used. Nevertheless, international standardisation of analysis and assessment of any potential biomarker is an important aspect for a successful translation into the routine setting [38].

In conclusion, Ki-67 is a biomarker for tumor cell proliferation. In our study, we found that a high Ki-67 expression was an independent prognostic marker for biochemical failure, high Gleason grade and larger tumor size. Despite unresolved issues on Ki-67 value cut-offs, we suggest that the analysis of Ki-67 add information regarding the aggressiveness of prostate tumors.

## Supporting information

S1 Table Minimal dataset with Ki-67 scoring. (SAV)

S1 Fig Comparison between the surgical centers. (PDF)
