# Supplementary material for: Evaluation of the proliferation marker Ki-67 in a large prostatectomy cohort
Source: PLoS One. 2017 Nov 15;12(11):e0186852. doi: 10.1371/journal.pone.0186852 (PMC5687762; doi:10.1371/journal.pone.0186852)
Supplement: S1 Fig — (PDF) [file pone.0186852.s002.pdf]

S1 Fig 1. Comparison between the surgical centers

Biochemical failure:

Overall Comparisons

Surg_center_comparison		Chi-Square	df	Sig.
1	Log Rank (Mantel-Cox)	1.876	1	.171
2	Log Rank (Mantel-Cox)	.254	1	**.615**

Test of equality of survival distributions for the different levels of diko_mean_1.34.

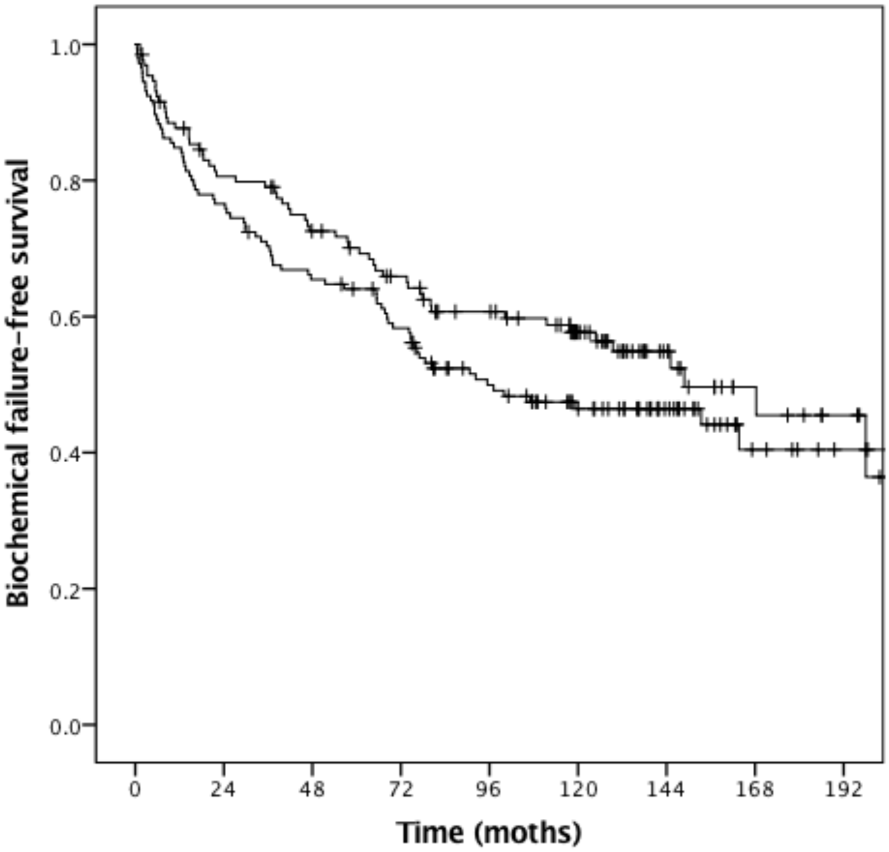

Clinical failure:

Overall Comparisons

Surg_center_comparison		Chi-Square	df	Sig.
1	Log Rank (Mantel-Cox)	.584	1	.445
2	Log Rank (Mantel-Cox)	.415	1	**.520**

Test of equality of survival distributions for the different levels of diko_mean_1.34.

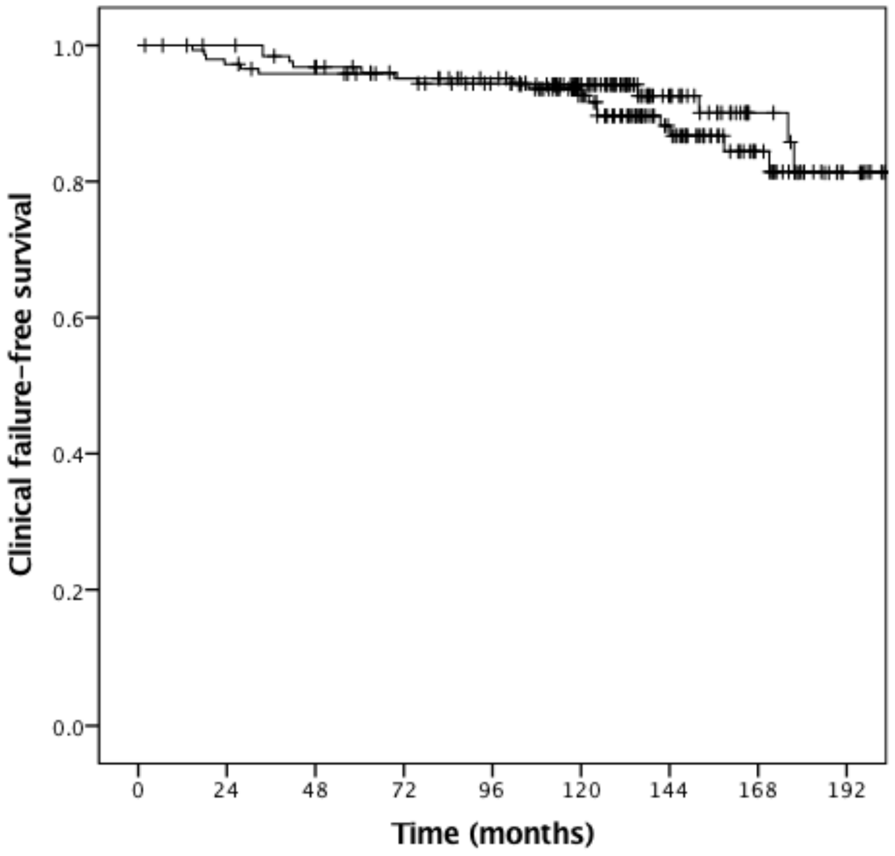

Prostate cancer death:

Overall Comparisons

Surg_center_comparison		Chi-Square	df	Sig.
1	Log Rank (Mantel-Cox)	.638	1	.424
2	Log Rank (Mantel-Cox)	2.747	1	**.097**

Test of equality of survival distributions for the different levels of diko_mean_1.34.

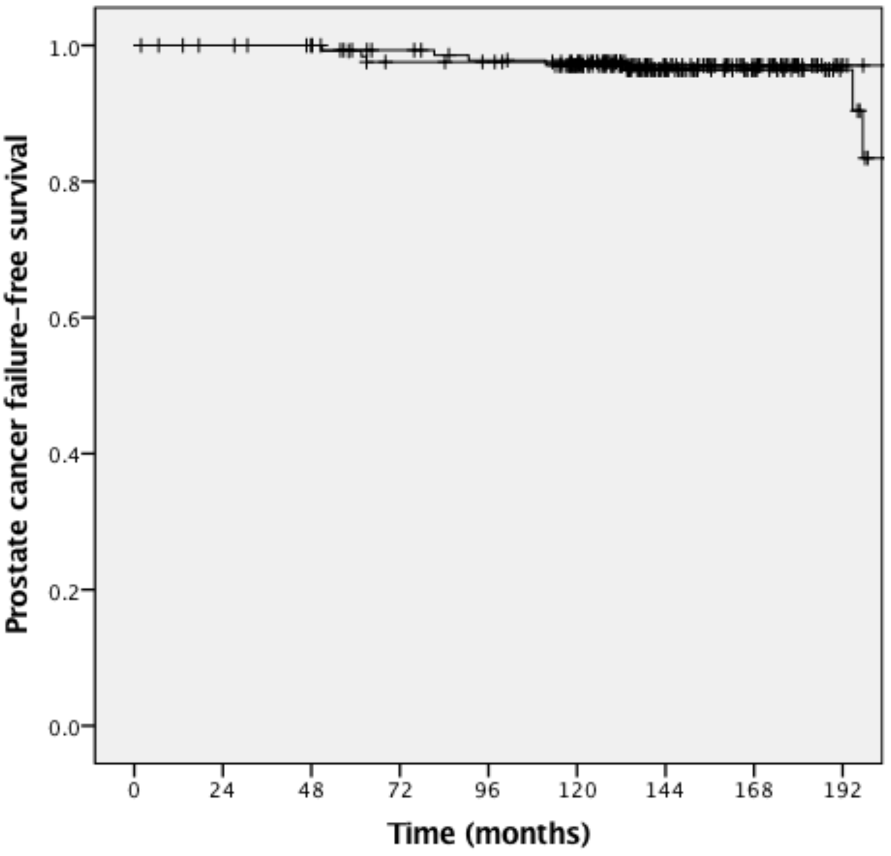

Biochemical failure:

Overall Comparisons

Surg_center_comparison		Chi-Square	df	Sig.
1	Log Rank (Mantel-Cox)	3.055	1	.080
2	Log Rank (Mantel-Cox)	.123	1	**.726**

Test of equality of survival distributions for the different levels of diko_mean_1.43.

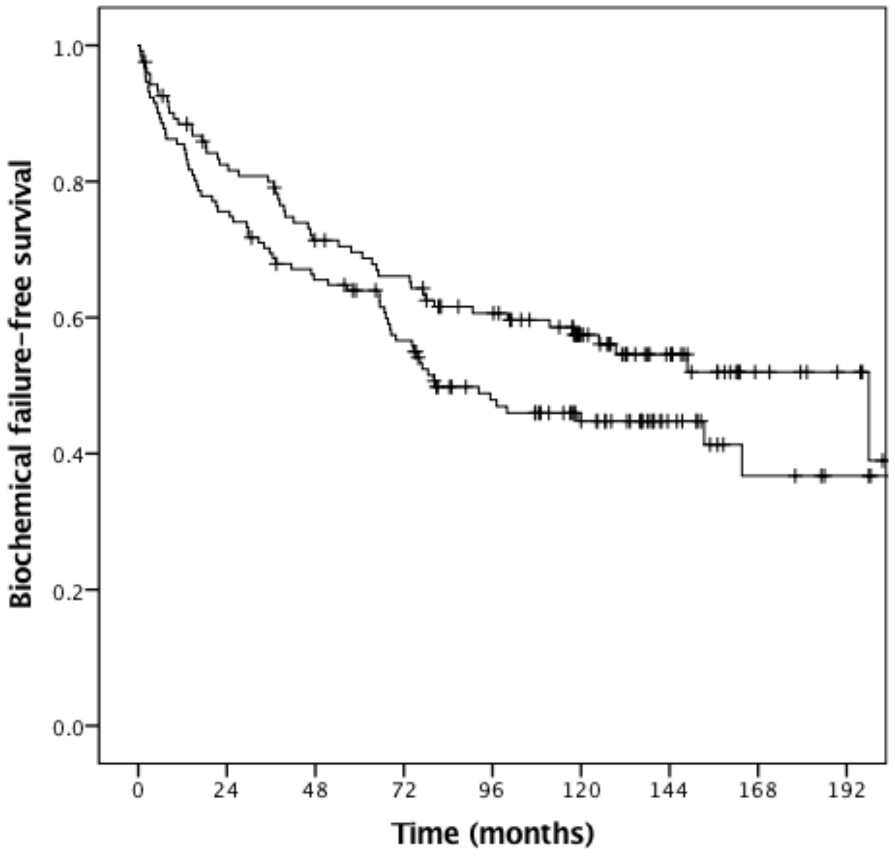

Clinical failure:

Overall Comparisons

Surg_center_comparison		Chi-Square	df	Sig.
1	Log Rank (Mantel-Cox)	2.835	1	.092
2	Log Rank (Mantel-Cox)	.236	1	**.627**

Test of equality of survival distributions for the different levels of diko_mean_1.43.

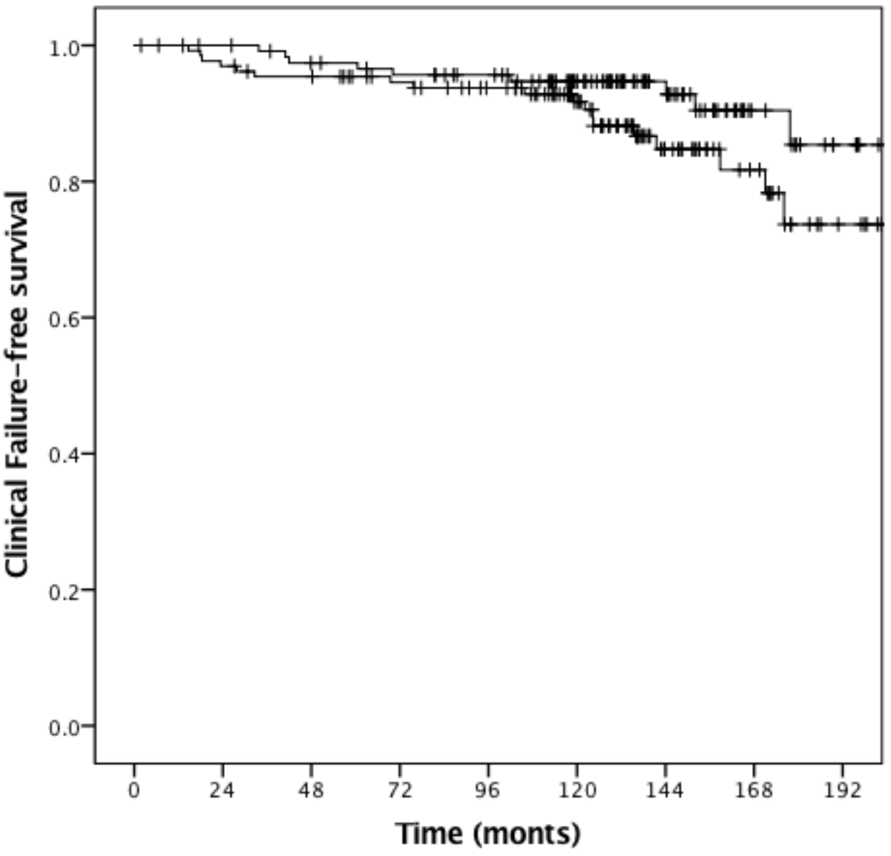

Prostate cancer death:
Overall Comparisons

Surg_center_comparison		Chi-Square	df	Sig.
1	Log Rank (Mantel-Cox)	.071	1	.790
2	Log Rank (Mantel-Cox)	.414	1	**.520**

Test of equality of survival distributions for the different levels of diko_mean_1.43.

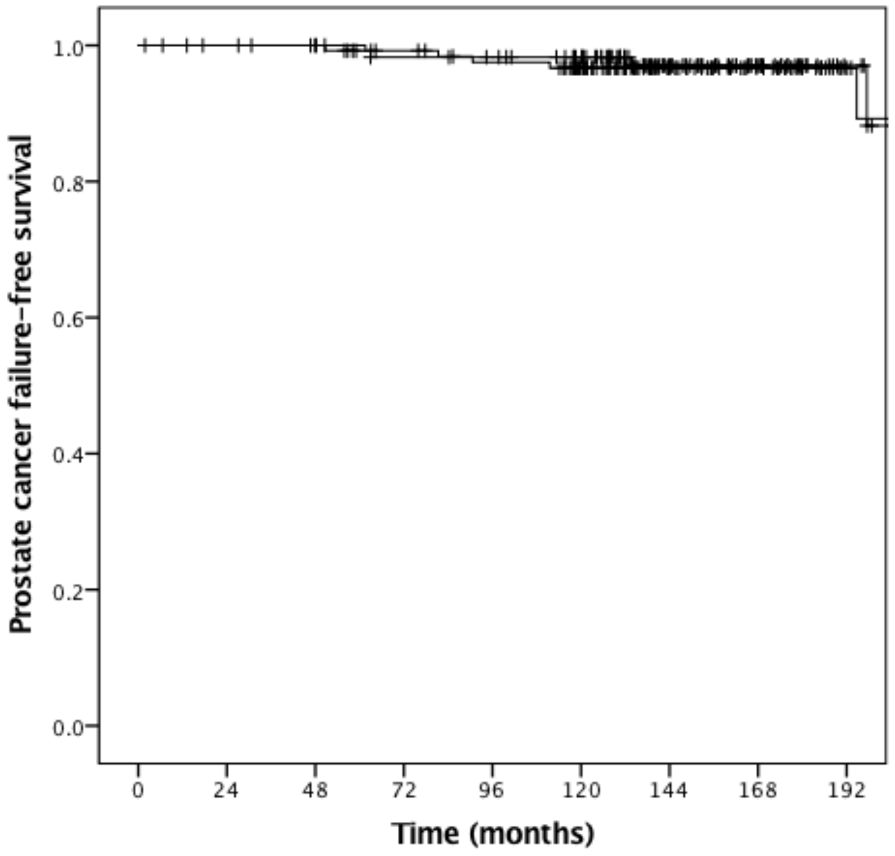

1.0
0.8
0.6
0.4
0.2
0.0
Prostate cancer failure-free survival
0
24
48
72
96
120
144
168
192
Time (months)
